# Human TLR1 Deficiency Is Associated with Impaired Mycobacterial Signaling and Protection from Leprosy Reversal Reaction

**DOI:** 10.1371/journal.pntd.0000231

**Published:** 2008-05-07

**Authors:** Elizabeth A. Misch, Murdo Macdonald, Chaman Ranjit, Bishwa R. Sapkota, Richard D. Wells, M. Ruby Siddiqui, Gilla Kaplan, Thomas R. Hawn

**Affiliations:** 1 University of Washington School of Medicine, Seattle, Washington, United States of America; 2 Mycobacterial Research Laboratory, Anandaban Hospital, Kathmandu, Nepal; 3 Laboratory of Mycobacterial Immunity and Pathogenesis, Public Health Research Institute at the University of Medicine and Dentistry of New Jersey, Newark, New Jersey, United States of America; René Rachou Research Center, Brazil

## Abstract

Toll-like receptors (TLRs) are important regulators of the innate immune response to pathogens, including *Mycobacterium leprae*, which is recognized by TLR1/2 heterodimers. We previously identified a transmembrane domain polymorphism, TLR1_T1805G, that encodes an isoleucine to serine substitution and is associated with impaired signaling. We hypothesized that this TLR1 SNP regulates the innate immune response and susceptibility to leprosy. In HEK293 cells transfected with the 1805T or 1805G variant and stimulated with extracts of *M. leprae*, NF-κB activity was impaired in cells with the 1805G polymorphism. We next stimulated PBMCs from individuals with different genotypes for this SNP and found that 1805GG individuals had significantly reduced cytokine responses to both whole irradiated *M. leprae* and cell wall extracts. To investigate whether TLR1 variation is associated with clinical presentations of leprosy or leprosy immune reactions, we examined 933 Nepalese leprosy patients, including 238 with reversal reaction (RR), an immune reaction characterized by a Th1 T cell cytokine response. We found that the 1805G allele was associated with protection from RR with an odds ratio (OR) of 0.51 (95% CI 0.29–0.87, p = 0.01). Individuals with 1805 genotypes GG or TG also had a reduced risk of RR in comparison to genotype TT with an OR of 0.55 (95% CI 0.31–0.97, p = 0.04). To our knowledge, this is the first association of TLR1 with a Th1-mediated immune response. Our findings suggest that TLR1 deficiency influences adaptive immunity during leprosy infection to affect clinical manifestations such as nerve damage and disability.

## Author Summary

*Mycobacterium leprae* (ML) causes a disabling and stigmatizing disease that is characterized by distinct immune responses. ML produces a spectrum of illness in humans, and several lines of evidence indicate that host genetic factors influence susceptibility and clinical manifestations. Leprosy can occur as the lepromatous or tuberculoid forms, which are associated with different clinical manifestations, histopathology, T cell cytokine profiles, and bacterial burden in affected sites. Leprosy is also associated with unique immunologic reactions, such as reversal reaction, which is characterized by the rapid development of a Th1 T cell cytokine response that can cause substantial morbidity. We and others recently discovered a common human polymorphism in TLR1 (T1805G, I602S) that regulates cytokine production in response to lipopeptide stimulation, influences the cellular innate immune response to *Mycobacteria*, is associated with altered localization, and is present in 50% of individuals worldwide. Here, we show that in humans the 1805G variant does not mediate an inflammatory response to ML *in vitro* and that this polymorphism is associated with protection from reversal reaction. These data suggest that a common variant of TLR1 is associated with altered adaptive immune responses to ML as well as clinical outcome.

## Introduction

*Mycobacterium tuberculosis* (MTb) has established latent infection in one-third of the world's population and, among those with progressive disease, causes about 2 million deaths per year [1]. *Mycobacterium leprae* (ML), a related organism, is the etiologic agent of leprosy, an ancient scourge that still causes illness in several regions of the world [2]. Both MTb and ML produce a spectrum of illness in their hosts, yet, aside from frank immunodeficiency, the host factors that underlie the various clinical manifestations of MTb and ML infection are largely unknown. One possible explanation for this diversity of outcomes is common, subclinical variation in host defense genes.

Several lines of evidence suggest that genetic factors influence susceptibility to leprosy and other *Mycobacteria* [1]–[5]. Rare individuals with primary immunodeficiency syndromes are highly susceptible to certain mycobacterial species due to Mendelian disorders associated with highly penetrant phenotypes [2]. However, in most individuals, susceptibility to mycobacterial infection is associated with complex inheritance patterns that are determined by the combined effects of variation across many genes, with a modest contribution from each polymorphism. Evidence that commonly inherited gene variants influence susceptibility to mycobacterial infection comes from twin studies, genome-wide linkage studies, and candidate gene association studies [2]. Studies of leprosy infection in twins have shown a three-fold greater concordance for type of leprosy disease in monozygotic compared to dizygotic twins [6]. Genome-wide linkage studies have identified two single nucleotide polymorphisms (SNPs) in the shared promoter region of the PARK2 and the PARCG gene, several HLA-DR2 alleles, and a non-HLA region near chromosome 10p13 that are associated with leprosy or leprosy subtypes [2],[7],[8]. Candidate gene association studies have also shown associations between leprosy and polymorphisms in several genes, including lymphotoxin-*a* (LTA) [9], the vitamin D receptor [10], TNF-α [11], laminin-2 [12], and mannose binding lectin [13],[14].

Human infection with *M. leprae* presents a unique opportunity to link innate and adaptive immune responses to host genetic factors. Leprosy's divergent clinical forms reflect two distinct immune responses to the same pathogen. Lepromatous leprosy (defined as polar lepromatous (LL) or borderline lepromatous (BL)) is characterized by a Th2 immune response and poor containment of the infection. At the opposite pole, tuberculoid leprosy (defined as polar tuberculoid (TT) or borderline tuberculoid (BT)) features a Th1 cytokine response, vigorous T cell responses to ML antigen, and containment of the infection in well-formed granulomas [15],[16]. Reversal reactions (RR) represent the sudden activation of a Th1 inflammatory response to ML antigens. They often occur after the initiation of treatment in patients towards the lepromatous pole of the leprosy spectrum (LL, BL, or borderline borderline (BB) categories) and reflect a switch from a Th2-predominant cytokine response toward a Th1-predominant response [15],[16]. Risk factors for RR intrinsic to the host include age [17] and gene variants, although the latter have not been intensively investigated [18],[19].

Toll-like receptors (TLRs) are a family of highly conserved, type 1 transmembrane proteins that orchestrate the innate immune response to microbial motifs, also known as pathogen associated molecular patterns (PAMPs) [20]–[22]. The TLR pathway regulates the innate immune response to mycobacteria through several TLRs, including TLR1, 2, 4, 6, and 9 [23]–[26]. TLR2 (Online Mendelian Inheritancce in Man (OMIM):603028), as a heterodimer with TLR1 (OMIM: 601194) or TLR6 (OMIM: 605403), mediates recognition of several mycobacterial motifs, including lipopeptides, the 19 kDa protein, lipoarabinomannan (LAM) [1],[27],[28]. Interaction of these ligands with the extracellular domain of TLRs leads to activation of a signaling pathway, which results in expression of chemokines and cytokines [20]. Functional work by many investigators has shown that TLR2 is a critical mediator of the innate immune response to ML and MTb [29],[30]. In addition, several TLR2 polymorphisms have been reported to be associated with susceptibility to MTb [31]–[33].

By contrast, very little is known about the effect of TLR1 variation on the innate response to mycobacteria or clinical susceptibility to mycobacterial disease. It also remains controversial whether and how the innate immune response mediated by any individual TLR shapes adaptive immunity [34]–[37]. We recently characterized a non synonymous SNP, T1805G (I602S), in the transmembrane domain of TLR1 that regulates signaling in response to PAM3, a synthetic ligand of TLR1 [38]. Johnson et al. also found that this polymorphism was associated with decreased signaling as well as protection from leprosy in Turkey [39]. Intriguingly, it appears that the TLR1 signaling defect is due to a complete absence of TLR1 on the surface of monocytes in GG individuals [39]. Here, we investigate an association of this SNP with different clinical forms of leprosy in Nepal and examine the effect of this SNP on leukocyte signaling in response to ML stimulation.

## Methods

### Materials

RPMI Medium 1640, L-glutamine, penicillin-streptomycin, and DMEM were from GIBCO/Invitrogen (Carlsbad, CA). Ultrapure lipopolysaccharide (LPS) was from *Salmonella minnesota* R595 (List Biological Labs, Inc.). Lipopeptides PAM2Cys-SKKKK (diacylated, PAM2) and PAM3Cys-SKKK (triacylated, PAM3) were from EMC Microcollections (Tuebingen, Germany). Macrophage-activating Lipopeptide-2 S-[2,3-*bis*(Palmityloxy)-(2*R*)-propyl-cysteinyl-GNNDESNISFKEK] (diacylated lipopeptide from *Mycoplasma fermentens*, Malp-2) was obtained from Alexis Biochemicals (Lausen, Switzerland). *M. leprae* reagents were obtained from J. Spencer (Colorado State University) through NIH, NIAID Contract No. NO1-AI-25469, entitled “Leprosy Research Support.” HEK293 cells (ATCC#CRL-1573) were grown in DMEM (GIBCO cat. no. #11995), supplemented with 10% fetal bovine serum, 10 units/ml penicillin, and 10 µg/ml streptomycin.

### Human Subjects and Study Design

Study participants in Seattle were healthy adults with no known history of unusual susceptibility to infections [40]. Study participants in Nepal included 933 leprosy patients referred for treatment at Anandaban Hospital in Katmandu, Nepal and later recruited to a study of genetic factors influencing susceptibility to reactional episodes in leprosy. The study population comprised more than 8 different ethnic and religious groups that included Brahmin (25.6%), Chhetri (22.3%), Tamang (14.3%), Newar (7.3%), Magar (5.4%), Muslim (3.3%), Sarki (3.5%), and Kami (2.7%), with 15.5% having unrecorded ethnicity A diagnosis of leprosy and determination of leprosy type was made by clinical symptoms, skin smears and biopsy reports. Assignment of leprosy category followed the Ridley/Jopling classification scheme [41]. Each patient had a minimum of three years of regular clinic follow-up prior to recruitment. In accord with guidelines of the US Department of Health and Human Services, protocols were approved by the Nepal Health Research Council, the University of Washington, the University of Medicine and Dentistry of New Jersey, and the Western Institutional Review Board. Written informed consent was obtained from all patients or from their relatives if the patient could not provide consent.

### Molecular Biology

DNA from subjects in Nepal was obtained by extraction from whole blood using Nucleon BACC2 Genomic DNA (Amersham Lifesciences) and Roche High-Pure PCR template preparation extraction kits. DNA from subjects in Seattle was extracted from whole blood using QIAamp DNA Blood Midi kits (Qiagen, Valenica, CA). Genotyping was carried out with a MassARRAY technique (Sequenom) as previously described [42],[43]. For functional studies, the coding region of TLR1 was amplified from genomic DNA and cloned into the pEF6/V5-His-TOPO vector (Invitrogen, Carlsbad, CA) as previously described [38]. To obtain the polymorphic variants of TLR1, a whole plasmid PCR strategy with mutant primers was used as previously described [30].

### Cytokine Assays

PBMCs were derived from whole blood separated by centrifugation on a Ficoll-Hypaque gradient, plated at a density of 1×10^5^ cells per well in 96-well plates in RPMI (supplemented with 10% fetal bovine serum), and incubated overnight. PBMC cytokine assays were then performed by stimulating with various TLR ligands or extracts of *M. leprae* for 18 hours. Each sample was assayed in triplicate. Cytokine levels were determined with a sandwich ELISA technique (Duoset, R&D Systems, Minneapolis, MN) or with a multiplex kit for the luminex platform (Human Fluorokine MAP Base Kit, Panel A, R&D systems, Minneapolis, MN). Levels of contaminating LPS as determined by the chromogenic *Limulus* amebocyte lysate test (Cambrex, MD) were 0.05–0.27, and 0.03–0.19 endotoxin units/ml, in wells incubated with whole irradiated ML (ML) and ML cell wall (MLcw), respectively, depending on the dose of reagent used. These values correspond to 4.5–27.2 and 3.04–19.4 pg/ml of endotoxin, respectively, in wells treated with whole irradiated ML and ML cw. All wells that received ML reagents were additionally treated with polymyxin B at a concentration of 10 µg/ml.

### Transfections

HEK293 cells were transfected with Polyfect (Qiagen, Hilden, Germany) per the manufacturer's instructions with 2–5×10^4^ cells per well in a 96-well plate with pRL-TK (to control for transfection efficiency), ELAM-luciferase (NF-κB reporter), one of two TLR1 variants, TLR2, and CD14. After an overnight transfection, cells were stimulated with TLR ligands or extracts of ML for 4–6 hours, and then lysed and processed for luciferase readings per the manufacturer's instructions for the Dual Luciferase Reporter Assay System (Promega, Madison, WI).

### Statistics

Univariate analysis was performed for categorical variables with a Chi-Square test; Fisher's exact test was used when the number of samples in a group was less than 5. The Mann-Whitney U-test was used to make comparisons of the cytokine production between groups, as small sample sizes precluded an assumption of normal distribution. Student's t-test was used to compare results in the luciferase assay. Two-sided testing was used for all comparisons to evaluate statistical significance. A P value (p) of ≤0.05 was considered significant. Statistics were calculated with Prism version 4.03software. For genetic analysis, allelic, genotypic, and haplotypic frequencies were compared between groups. Haplotypes were constructed with an Expectation/Maximization (EM) algorithm with the program HAPIPF in IC Stata (version 10.0) [44]. Except for minor deviations, the observed allelic frequencies of SNPs were consistent with expected frequencies under Hardy-Weinberg equilibrium.

## Results

### TLR1 SNP T1805G regulates signalling in response to *M. leprae* in transfected cells

**Figure 1:**
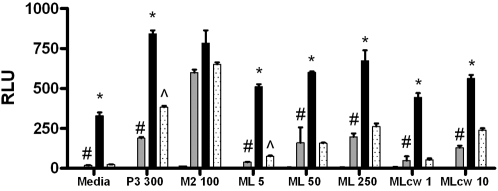
NF-κB activity in response to *M. leprae* is diminished in the 1805T variant. HEK293 cells were transfected with an NF-κB luciferase reporter, a *Renilla* luciferase construct to control for transfection efficiency (pRL-TK), and CD14. Additional transfectants varied by condition and included: empty plasmid vector (EV, clear), TLR2 alone (T2, gray), or TLR2 with one of two TLR1 constructs, 1805T (black) or 1805G (stippled). Luciferase activity represents basal (media stimulation) or stimulated activity of transfected cells. Mean values (+/−standard deviation) are depicted for two representative experiments, each performed in triplicate. RLU, relative luciferase units; P3 300, PAM3 at a dose of 300 ng/ml; M2 100, Malp-2, at 100 ng/ml; ML, whole, irradiated ML at 5, 50, or 250 µg/ml; MLcw, *M. leprae* cell wall at a dose of 1 or 10 µg/ml. * = P≤0.01, by Student's t-test when comparing the 1805T and 1805G variants (both with T2); # = P≤0.01, by Student's t-test when comparing T2+1805T and T2; ˆ = P≤0.01, by Student's t-test when comparing T2+1805G and T2.

We investigated the effect of SNP T1805G (I602S) on NF-κB responses to ML in HEK293 cells transfected with 1805T (602I) or 1805G (602S), firefly luciferase conjugated to an NF-κB promoter (ELAM), TLR2, CD14, and *Renilla* luciferase conjugated to thymidine kinase to control for transfection efficiency [38]. Cells were then stimulated with media, ML extracts or TLR ligands: PAM3, a ligand for the TLR2/1 heterodimer or Malp-2, a ligand for the TLR2/6 heterodimer (Fig 1). HEK293 cells transfected with TLR2+1805T and stimulated with 50 µg/ml of whole, irradiated ML had significantly greater NF-κB activity than HEK293 cells transfected with TLR2 alone (600.7 vs. 159.8 relative luciferase units (RLU), p = 0.001), or cells transfected with TLR2+1805G (600.7 vs. 157.1 RLU, p = 0.000004) (Fig 1). Responses to ML were dose-dependent in cells transfected with either TLR1 variant and the signaling difference between 1805T and 1805G transfectants persisted over a range of doses (comparison for ML 5 µg/ml: 511.9 vs. 75.8, p<0.0001; comparison for ML 250 µg/ml: 673.7 vs. 262.8, p = 0.0005). We then investigated whether T1805G influenced signaling in response to MLcw, which contains lipopeptide moieties known to stimulate through TLR2/1 [29]. TLR2+1805T-transfected cells stimulated with 1 or 10 µg/ml of MLcw had significantly greater NF-κB activity than cells transfected with TLR2+1805G (Fig 1, comparison for MLcw 1 µg/ml: 444.4 vs. 53.8 RLU, p = 0.00005; comparison for MLcw 10 µg/ml: 562.8 vs. 238.1 RLU, p = 0.004). As a control, we also compared baseline signalling activity and response to tri-acylated lipopeptide (PAM3) in the two 1805 variants. Consistent with previous observations [38], the 1805T variant, when co-transfected with TLR2, mediated greater constitutive NF-κB activity compared to TLR2 alone (Fig 1, stimulation with media alone: 329.7 vs. 3.9 RLU, p = 0.000003). The TLR2+1805T transfectants were also readily distinguished from the TLR2+1805G transfectants by a significantly higher level of basal signaling (329.7 vs. 24.2 RLU, p = 0.000002). In addition, TLR2+1805T-transfected cells stimulated with PAM3 had significantly greater NF-κB activity compared to cells transfected with TLR2+1805G (841.4 vs. 383.7 RLU, p = 0.0009). In contrast, responses to Malp-2 did not significantly differ between the two variants (783.1 vs. 650.6 RLU, p = 0.37). Together, these results suggest that TLR1 variant 1805G leads to impaired innate immune responses to ML because of a defect in basal signaling of the TLR2/1 heterodimer.

### TLR1 SNP T1805G regulates the inflammatory response to *M. leprae* in human monocytes

**Figure 2:**
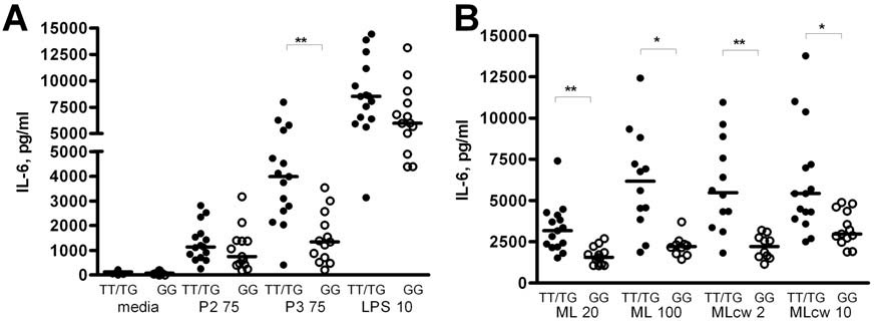
IL-6 production by human primary cells following stimulation with *M. leprae.* Peripheral blood mononuclear cells were stimulated for 18 hours and supernatants were assayed for cytokine production by ELISA. PBMCs were derived from whole blood taken from 15 individuals with the genotype 1805TTor 1805TG (TT/TG: dark circles) and 13 individuals with the 1805GG genotypes (GG: open circles). (A): PBMCs stimulated in triplicate with media, PAM2 at 75 ng/ml (P2 75), PAM3 at 75 ng/ml (P3 75), or LPS at 10 ng/ml (LPS 10). (B): PBMCs stimulated in triplicate with whole irradiated ML at 20 or 100 µg/ml (ML20 or ML100) or MLcw at 2 or 10 µg/ml (MLcw 2 or MLcw10). The mean level and standard error of the mean are depicted and were derived from averaging the responses of individuals stimulated in triplicate. The median level is depicted by a bar. *P≤0.01, **P≤0.001 by Mann–Whitney U-test.

**Figure 3:**
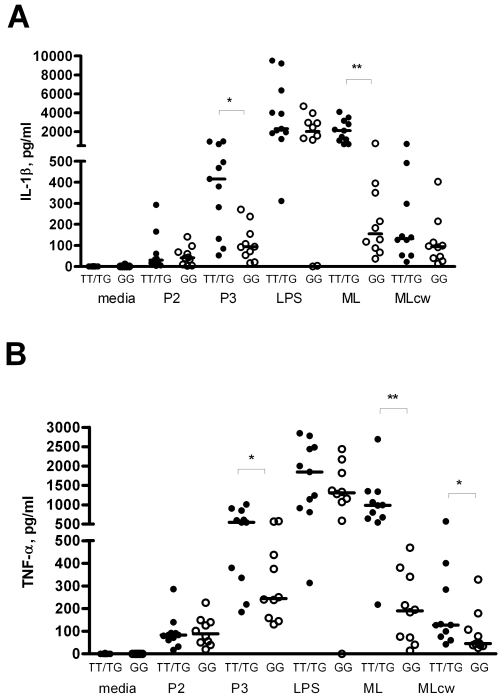
IL-β and TNF-α production in human mononuclear cells following stimulation with *M. leprae*. Peripheral blood mononuclear cells were stimulated for 18 hours and supernatants were assayed for cytokine production by luminex multiplex bead assay. PBMCs were obtained from 11 individuals with the genotype 1805TT or 1805TG (TT/TG: dark circles) and 10 individuals with the 1805GG genotypes (GG: open circles). PBMCs stimulated with media, PAM2 at 75 ng/mL (P2), PAM3 at 75 ng/mL (P3), or LPS at 10 ng/ml (LPS), whole irradiated ML at 20 µg/ml (ML) or MLcw at 10 µg/ml (MLcw). IL-1β production following stimulation is shown in (A), and TNF-α production in (B). The mean level and standard error of the mean are depicted and were derived from averaging the responses of individual within each genotype group. The median level is depicted by a bar; *p≤0.05, **p≤0.001 by Mann–Whitney U-test.

We next examined whether TLR1_T1805G regulates innate immune responses to ML in human primary immune cells. We obtained PBMC from whole blood from 28 healthy individuals whose genotypes for TLR1_T1805G had previously been determined [38]. The PBMCs were then stimulated with whole, irradiated ML, MLcw, and a variety of TLR ligands, including PAM3, PAM2, and LPS. We compared the high (1805TT) and medium (1805TG) responding genotypes with the low responding genotype (1805GG) (Fig 2). When stimulated with MLcw, PBMCs from 1805TT or 1805TG (1805TT/TG) donors showed significantly greater IL-6 responses compared to 1805GG PBMCs (Fig 2B, for MLcw at 2 µg/ml: 5,950 vs. 2,198 pg/ml, p = 0.0005; for MLcw at 10 µg/ml: 6,115 vs. 3,320 pg/ml, p = 0.0076). Similarly, responses to whole, irradiated ML were significantly higher in 1805TT/TG PBMCs compared to 1805GG PBMCs (Fig 2B, for whole, irradiated ML at 20 µg/ml: 3,310 vs. 1,649 pg/ml, p = 0.0005; for whole, irradiated ML at 100 µg/ml: 6,183 vs. 2,246 pg/ml, p = 0.0017). As previously observed, after stimulation with PAM3, significantly higher levels of IL-6 were seen in PBMCs heterozygous or homozygous for 1805T compared to1805GG PBMCs (Fig 2A, for TT/TG genotypes vs. GG genotypes stimulated with 75 µg/mL PAM3: 3,966 vs. 1,491 pg/ml, p = 0.0007). Stimulation with LPS and PAM2, ligands with specificity for TLR4 and TLR2/6, respectively, produced no significant differences in IL-6 production between the two groups (Fig 2A). We also assessed the levels of other cytokines important in the monocyte immune response to mycobacteria and found that IL-1β production in PBMCs from 1805TT/TG donors stimulated with PAM3 or whole irradiated ML was significantly higher than in 1805GG PBMCs (Fig 3A). IL-1β levels did not differ between the two groups when PBMCs were stimulated with PAM2 or with LPS (TLR2/6 and TLR4 ligands, respectively) controls. Production of TNF-α, similarly, was significantly higher in 1805TT/TG PBMCs stimulated with PAM3, whole irradiated ML, or MLcw compared to 1805GG PBMCs (Fig 3B), but did not differ between the two groups after stimulation with Pam2 or LPS (TLR2/6 and TLR4 controls) (Fig 3B). Interestingly, there were no differences in IL-1β levels between TT/TG and GG groups after PBMC stimulation with MLcw, in contrast to the pattern seen with other cytokines (Fig 2B, 3A).

### TLR1 is not associated with altered risk of tuberculoid or lepromatous forms of leprosy

Although recently published data suggests that TLR1_T1805G is associated with susceptibility to leprosy, associations with different types of leprosy or immunologic reactions have not been previously examined. To determine whether the TLR1_T1805G polymorphism was associated with different forms of leprosy or leprosy immune reactions, 933 patients from Anandaban Hospital in Kathmandu, Nepal were enrolled in a retrospective study. Of this total, 581 had polar lepromatous (LL), borderline leprosy (BL) or borderline borderline (BB) and 343 had tuberculoid leprosy (including borderline tuberculoid (BT) and polar tuberculoid (TT)). A total of 344 patients experienced immune reactions during 3 years of regular visits to a leprosy clinic, of whom 238 had RR and 108 had erythema nodosum leprosum (ENL) and 2 had both reactions. The baseline characteristics of this population are described in Table 1. When individuals with lepromatous leprosy were compared to those with tuberculoid leprosy, there were no significant associations between SNP T1805G and either form of leprosy, either at the allelic or genotypic level of analysis (Table 2). However, there was a trend toward an association of the TG or GG genotypes with lepromatous leprosy (OR [odds ratio] 4.76, 95% CI [95% confidence interval] 0.58–38.87, p = 0.11) in comparison to tuberculoid leprosy that did not reach significance. We also genotyped five additional TLR1 SNPs that are contained in common TLR1 haplotypes. We did not find associations of any of these SNPs with leprosy type (Table 2). Analysis of TLR1 haplotypes generated from these six SNPs similarly yielded no association with leprosy type (data not shown).

**Table 1:** Baseline characteristics of leprosy subjects.

Characteristics, n (%)	All leprosy^1^	Brahmin^2^	Chhetri	Tamang	Newar	*P* 3
	933 (100.0)	239 (25.6)	208 (22.3)	134 (14.3)	68 (7.3)	
Gender						
female	280 (30.0)	73 (30.5)	61 (29.3)	44 (32.8)	22 (32.4)	0.92
male	649 (69.6)	166 (69.5)	146 (70.2)	90 (67.2)	46 (67.6)	
Age (mean+/−SD)	44.2±16.4	45.5±15.4	43.6±16.7	44.1±16.7	51.4±17.1	
Leprosy type^2^						
Lepromatous (LL, BL)	551 (59.1)	126 (52.7)	122 (58.7)	89 (66.4)	38 (55.9)	0.26
Borderline (BB)	30 (3.2)	11 (4.6)	5 (2.4)	6 (4.5)	2 (2.9)	
Tuberculoid (TT, BT)	343 (36.8)	96 (40.2)	80(38.5)	39 (29.1)	27 (39.7)	
Immune Reactions						
No RR	695 (74.5)	185 (77.4)	149 (71.6)	89 (66.4)	54 (79.4)	0.07
RR	238 (25.5)	54 (22.6)	59 (28.4)	45 (33.6)	14 (20.6)	
No ENL	442 (80.4)	104 (83.2)	106 (86.9)	73 (82.0)	34 (89.5)	0.61
ENL	108 (19.6)	21 (16.8)	16 (13.1)	16 (18.0)	4 (10.5)	

1 Percentages of individuals in some categories do not sum to 100 due to missing data. Not shown among all leprosy are 8 subjects with peripheral neuropathy (PN), and one with leprosy of indeterminate type (IN).

2 The four most frequent ethnic groups (from > 8 ethnic groups) are tabulated.

3 P represents exact *P* values for overall distribution of gender and age groups within the different ethnic groups and for the overall distribution of leprosy type and immune reactions among the 4 different ethnic groups.

**Table 2:** TLR1 Polymorphism Frequency in Different Leprosy Types.

SNP	Allele Frequency (%)		OR (95% CI)	P^1^	Genotype frequency (%)			
	*A* 3	*a*			*AA*	*Aa*	*aa*	P^2^
**Trs5743563C**								
*Tuberculoid* 4	510 (78.0)	144 (22.0)			196 (59.9)	118 (36.1)	13 (4.0)	
*Lepromatous*	825 (75.1)	273 (24.9)	1.17 (0.93–1.46)	0.18	306 (55.7)	213 (38.8)	30 (5.5)	0.38
**Ars5743565G**								
*Tuberculoid*	505 (77.7)	145 (22.3)			194 (59.7)	117 (36.0)	14 (4.3)	
*Lepromatous*	829 (75.2)	273 (24.8)	1.15 (0.91–1.44)	0.24	308 (55.9)	213 (38.7)	30 (5.4)	0.49
**Trs5743592C**								
*Tuberculoid*	493 (75.6)	159 (24.4)			186 (57.1)	121 (37.1)	19 (5.8)	
*Lepromatous*	791 (72.8)	295 (27.2)	1.16 (0.93–1.45)	0.20	286 (52.7)	219 (40.3)	38 (7.0)	0.43
**Trs5743595C**								
*Tuberculoid*	508 (77.9)	144 (22.1)			195 (59.8)	118 (36.2)	13 (4.0)	
*Lepromatous*	833 (75.6)	269 (24.4)	1.14 (0.91–1.44)	0.27	310 (56.3)	213 (38.7)	28 (5.1)	0.52
**G743A**								
*Tuberculoid*	359 (55.6)	287 (44.4)			107 (33.1)	145 (44.9)	71 (22.0)	
*Lepromatous*	604 (55.7)	480(44.3)	0.99 (0.82–1.21)	0.95	172 (31.7)	260 (48.0)	110 (20.3)	0.67
**T1805G**								
*Tuberculoid*	586 (94.2)	36 (5.8)			276 (88.7)	34 (10.9)	1 (0.3)	
*Lepromatous*	912 (93.1)	68 (6.9)	1.21 (0.80–1.84)	0.36	430 (87.8)	52 (10.6)	8 (1.6)	0.23

1 P value for comparison of allele frequencies.

2 P value for comparison of genotype frequencies.

3 ***A*** denotes common allele, ***a*** denotes minor allele.

4 Tuberculoid includes TT and BT. Lepromatous includes LL, BL, and BB.

### TLR1 variant 1805G is associated with protection from reversal reaction

We next investigated whether T1805G (I602S) might be associated with ENL or RR, a Th1-mediated immune event clinically manifested by inflamed skin lesions, fever, and neuritis. There was no association of T1805G or any other TLR1 polymorphisms with ENL. In contrast, the 1805G allele was associated with a reduced risk of developing RR in comparison to the 1805T allele (Table 3). The allele frequency of 1805G was 3.9% in those with RR versus 7.4% in those without (unadjusted OR 0.51, 95% CI 0.29–0.87, p = 0.01). The distribution of the genotype frequencies was also significantly different with a p value of 0.05 (Table 3). We next examined whether this association was affected by population admixture. This cohort contains representatives of more than 8 different ethnic groups with the majority belonging to one of four groups (Brahmin, Chhetri, Tamang, and Newar). There were no significant differences in frequencies of leprosy type or immunologic reactions among the different ethnic groups (Table 1). We performed a multivariate logistic regression, adjusting for ethnicity, and found that the odds ratio remained significant (OR 0.52, 95% CI 0.30–0.92, p = 0.03). We also adjusted for ethnicity, sex and age as a continuous variable, and again found that the odds ratio remained significant (OR 0.54, 95% CI, 0.30–0.96, p = 0.04).

**Table 3:** TLR1 Polymorphism Frequency in Reversal Reaction.

SNP	Allele Frequency (%)		OR (95% CI)	P^1^	Genotype frequency (%)			P^2^
	A^3^	a			AA	Aa	aa	
**Trs5743563C**								
No reaction	1014 (77.3)	298 (22.7)	1.00		388 (59.1)	238 (36.3)	30 (4.6)	
Reaction	335 (73.5)	121 (26.5)	1.23 (0.96–1.57)	0.10	120 (52.6)	95 (41.7)	13 (5.7)	0.22
**Ars5743565G**								
No reaction	1013 (77.1)	301 (22.9)	1.00		388 (59.1)	237 (36.1)	32 (4.9)	
Reaction	335 (73.8)	119 (26.2)	1.20 (0.94–1.53)	0.15	120 (52.9)	95 (41.9)	12 (5.3)	0.26
**Trs5743592C**								
No reaction	984 (75.2)	324 (24.8)	1.00		371 (56.7)	242 (37.0)	41 (6.3)	
Reaction	313 (70.2)	133 (29.8)	1.29 (1.02–1.64)	**0.04**	106 (47.5)	101 (45.3)	16 (7.2)	0.06
**Trs5743595C**								
No reaction	1017 (77.4)	297 (22.6)	1.00		389 (59.2)	239 (36.4)	29 (4.4)	
Reaction	338 (74.1)	118 (25.9)	1.20 (0.94–1.53)	0.16	122 (53.5)	94 (41.2)	12 (5.3)	0.32
**G743A**								
No reaction	710 (54.7)	588 (45.3)	1.00		202 (31.1)	306 (47.1)	141 (21.7)	
Reaction	262 (58.5)	186 (41.5)	0.86 (0.69–1.07)	0.17	80 (35.7)	102 (45.5)	42 (18.8)	0.39
**T1805G**								
No reaction	1117 (92.6)	89 (7.4)	1.00		523 (86.7)	71 (11.8)	9 (1.5)	
Reaction	396 (96.1)	16 (3.9)	**0.51** (0.29–0.87)	**0.01**	190 (92.2)	16 (7.8)	0 (0.0)	**0.05** 4

1 P value for comparison of allele frequencies by Chi-square.

2 P value for comparison of genotype frequencies calculated by Chi-square unless otherwise indicated.

3 ***A*** denotes common allele, ***a*** denotes minor allele.

4 P value calculated by Fisher's Exact test due to small cell number.

Previously, we found that TG individuals are intermediate between TT and GG individuals in responses to TLR1 stimulation [38]. We therefore investigated the influence of TG heterozygotes with a recessive (assumes T is recessive to G and compares TT versus TG/GG frequencies) or a dominant model (assumes T is dominant over G and compares TT/TG versus GG frequencies). In the recessive model, we found that the 1805 TG/GG genotypes were associated with a lower likelihood of RR compared to the TT genotype (OR 0.55, 95% CI 0.31–0.97, p = 0.04). In the dominant model, the GG genotype was associated with a non-significant reduction in risk when compared to the TT/TG genotypes (OR 0.15, 95%CI 0.01–2.62, p = 0.12).

We next examined other TLR1 polymorphisms to determine whether there were any additional associations between individual SNPs or haplotypes and RR. SNP rs5743592, located in intron 2 adjacent to the 5′ UTR of TLR1, was found to be associated with a modestly increased risk of RR (Table 3, OR for allelic comparison: 1.29, 95% CI 1.02–1.64, p = 0.04). When haplotypes of 6 TLR1 SNPs were examined (Table 4), one haplotype, TATTAG, was associated with protection from RR (OR 0.55, 95% CI 0.31–0.97, p = 0.05). None of the other five haplotypes had any association with RR. Haplotype TATTAG was the only haplotype occurring with a frequency greater than 1% that contained the 1805G allele. Lastly, we examined whether haplotypes formed from SNPs rs5743592 and 1805 were associated with altered risk of RR. The haplotype associations were consistent with the individual effect of each SNP on the risk RR (Table 4), without any additive or synergistic effects. Together, these genetic data demonstrate that TLR1 SNP 1805G is associated with protection from RR.

**Table 4:** Frequency of TLR1 haplotypes in reversal reaction.

6 SNP Haplotypes^1^	Reversal Reaction		OR (95% CI)	P^3^
	No	Yes		
TATTAT	441 (37.8)	155 (38.1)	1.00	
TATTGT	356 (30.5)	116 (28.5)	0.93 (0.70–1.22)	0.68
CGCCGT	259 (22.2)	105 (25.8)	1.15 (0.86–1.54)	0.46
**TATTAG**	82 (7.1)	16 (3.9)	**0.55** (0.31–0.97)	**0.05**
TACTGT	26 (2.2)	15 (3.7)	1.64 (0.85–3.17)	0.15
CGCCGG	2 (0.1)	0 (0.0)	0.25 (0.00–51.20)	0.58
**2 SNP Haplotypes** 2				
TT	808 (68.0)	272 (66.4)	1.00	
CT	294 (24.7)	122 (29.7)	1.23 (0.95–1.58)	0.18
**TG**	83 (7.0)	16 (3.8)	**0.56** (0.32–0.97)	**0.05**
CG	3 (0.3)	0 (0.1)	0.41 (0.02–10.61)	0.58

1 Order for 6 SNP haplotypes, left to right: Trs5743563C, Ars5743565G, Trs5743592C, Trs5743595C, G743A, T1805G.

2 Order for 2 SNP haplotypes: Trs5743592C, T1805G.

3 P value represents comparison of a given haplotype with the reference haplotype TATTAT.

## Discussion

In this manuscript, we demonstrate that a human TLR1 SNP regulates the innate immune response to ML and is associated with protection from RR. This is the first study to describe an association of a TLR1 SNP with a Th1-mediated adaptive immune response. One weakness of our study is the low frequency of the 1805G variant in Nepal, which limited our power to detect associations with leprosy type. However, the relevance of the 1805G SNP to leprosy pathogenesis is supported by work from Johnson and colleagues, who recently reported an association of this variant with protection from leprosy in a Turkish cohort [39]. Although these authors do not mention whether or not T1805G was associated with different forms of leprosy in Turkey or with immune reactions, this may be due to the small size of their patient cohort (57 individuals). Genetic association studies that utilize cohorts of multiple ethnicities are also open to the criticism that associations are due to the effects of population admixture rather than the variant of interest. However, when we adjusted for ethnic composition of the comparision groups, we still found that the 1805G variant was associated with significant protection against RR.

There are several possible mechanisms by which TLR1 might affect the pathogenesis of RR. At the cellular level, TLR1 might exert its influence through control of innate immune functions, such as the capacity of dendritic cells (DCs) and macrophages to control bacillary replication. Alternatively, or in addition, SNP 1805 may regulate DC maturation and/or antigen presentation and thereby influence the activation and maintenance of T cell responses to *M. leprae* antigens. Interestingly, recent work by other investigators demonstrates that the differentiation of monocytes into mature antigen presenting cells (APCs) is mediated by TLR signaling [35],[36]. For example, the 19 kDa protein of MTb signals through TLR2/1 to downregulate MHC class I and II antigen presentation by macrophages, leading to impaired T cell activation [45]. We have previously shown that ML also exerts an inhibitory effect on APC activation and maturation, through an as-yet unidentified mechanism [46]. In LL patients who are clinically stable, macrophages within LL lesions contain numerous bacilli that are seemingly resistant to host killing [47],[48]. However, this inhibition of phagocyte function seems to be overturned during RR. When patients undergo RR, the bacilli within these macrophages are rapidly cleared. This clearance coincides with the influx into the lesion of CD1b+ DC, which activate *M. leprae*-specific T cells and thereby promote intracellular killing. Importantly, the generation of CD1b+ DCs appears to be dependent on signaling through TLR2/1 [48]. Here, we show that individuals carrying the 1805G SNP are protected against reversal reactions. Our data suggests that TLR1 may be an important regulator of these effects on DCs.

At the molecular level, the defect in 1805G signaling is likely due to a failure to express or retain TLR1 on the cell surface. Johnson recently showed that monocytes from 1805GG individuals completely lack surface TLR1, although total levels of this receptor are normal [39]. In Nepal, we observed a trend toward an association of the 1805G variant with lepromatous rather than tuberculoid leprosy. This trend, although not statistically significant, is consistent with impaired Th1 immunity, which is required for the tuberculoid form of the disease. An association of 1805G with lepromatous leprosy could explain the intriguing earlier observation by Krutzik and coworkers, who examined TT and LL lesions and were unable to detect any TLR1 staining in LL lesions [29]. Collectively, these findings suggest that TLR1 biology is different in lepromatous leprosy than in other forms of the disease. The absence of membrane-inserted TLR1 in 1805GG individuals may be associated with a Th2 immune response that arises by default in the absence of robust Th1 cytokine responses. This Th2 bias may in turn permit continued replication of the *M. leprae* bacillus and result in the clinical phenotype of LL.

In our initial characterization of TLR1_T1805G, we found that this polymorphism is present in up to 76% of Caucasian Americans and is associated with a defect in innate responses to bacterial lipopeptide [38]. Worldwide, the T1805G polymorphism has variable frequency across ethnic groups. In Turkey, the allele frequency of this variant is 43% [39], while among African Americans and Vietnamese individuals, it has a frequency of 25% and 1%, respectively [38]. A broad array of pathogens are sensed by TLR2, and consequently by TLR2/1 or TLR2/6 heterodimers. These microorganisms include gram-positive and gram-negative bacteria, fungi, parasites, and mycobacteria [49]. Given the association of TLR1 1805G with Th1-mediated immune events, this SNP may influence the pathogenesis of any number of inflammatory conditions, including chronic mycobacterial infection, autoimmune disorders, sepsis and allergic reactions.
